# Supplementary material for: Heat adaptation of phage T7 under an extended genetic code
Source: Virus Evol. 2021 Dec 1;7(2):veab100. doi: 10.1093/ve/veab100 (PMC8923235; doi:10.1093/ve/veab100)
Supplement: veab100_Supp [file veab100_supp.zip › Sequencing_Analysis.zip!/Sequencing_Analysis/S3/evidence/RA_33.html]

BRESEQ :: Evidence


| Read alignment evidence... | | | | | | | | | | | |
| --- | --- | --- | --- | --- | --- | --- | --- | --- | --- | --- | --- |
|  | seq id | position | | ref | new | freq | score (cons/poly) | reads | annotation | genes | product |
| \* | NC\_001604.1 | 29,691 | 0 | A | G | 63.9% | 1272.5 / inf | 1475 | intergenic (–/–) | *–/–* | –/– |
| Reads supporting (aligned to +/- strand):  **ref** base A (252/276);  **new** base G (430/503);  **total** (691/784) | | | | | | | | | | | |
| Fisher's exact test for biased strand distribution *p*-value = 5.49e-01 | | | | | | | | | | | |
| Kolmogorov-Smirnov test that lower quality scores support variant *p*-value = 8.58e-01 | | | | | | | | | | | |

Only 100 of 1486 total aligned reads displayed.  

`AGGAACAAGTTCAGAATCAGATGAACGCATGGACGAAAGCTCAGGCCAAGGCTCTGGACGATTCCATGAAGTCAATGAACAAACTTGACGTAATCGACAAGCAATTCCAGAAGCGAATCAACGGTGAGTGGGTCTCAACGGATTTTAAGGATATGCCAGTCAACGAGAACACTGGTGAGTTCAAGCATAGCGATATGGTTAACTACGCCAATAAGAAGCTCGCTGAGATTGACAGTATGGACATTCCAGACGGTGCCAAGGATGCTATGAAGTTGAAGTACCTTCAAGCGGACTCTAAGGACGGAGCATTCCGTACAGCCATCGGAACCATGGTCACTGACGCTGGTCAAGAGTGGTCTGCCGCTGTGATTAACGGTAAGTTACCAGAACGAACCCCAGCTATGGATGCTCTGCGCAGAATCCGCAATGCTGACCCTCAGTTGATTGCTGCGCTATACCCAGACCAAGCTGAGCTATTCCTGACGATGGACATGATGGACAAGCAGGGTATTGACCCTCAGGTTATTCTTGATGCCGACCGACTGACTGTTAAGCGGTCCAAAGAGCAACGCTTTGAGGATGATAAAGCATTCGAGTCT  >  NC_001604.1/29391‑29989`  

                                                                                                                                                                                                                                                                                                            `|`                                                                                                                                                                                                                                                                                                            
`aGGAACAAGTTCAGAATCAGATGAACGCATGGACGAAAGCTCAGGCCAAGGCTCTGGACGATTCCATGAAGTCAATGAACAAACTTGACGTAATCGACAAGCAATTCCAGAAGCGAATCAACGGTGAGTGGGTCTCAACGGATTTTAAGGATATGCCAGTCAACGAGAACACTGGTGAGTTCAAGCATAGCGATATGGTTAACTACGCCAATAAGAAGCTCTCTGAGATTGACAGTATGGACATTCCAGACGGTTCCAAGGATGCTATGAAGTTGAAGTACCTTCAATCGGACTCTAAGGa                                                                                                                                                                                                                                                                                                            <  1:247657/301‑1 (MQ=255)`  
`aGGAACAAGTTCAGAATCAGATGAACGCATGGACGAAAGCTCAGGCCAAGGCTCTGGACGATTCCATGAAGTCAATGAACAAACTTGACGTAATCGACAAGCAATTCCAGAAGCGAATCAACGGTGAGTGGGTCTCAACGGATTTTAAGGATATGCCAGTCAACGAGAACACTGGTGAGTTCAAGCATAGCGATATGGTTAACTACGCCAATAAGAAGCTCACTGAGATTGACAGTATGGACATTCCAGACGGTGCCAAGGATGCTATGAAGTTGAAGTACCTTCAAGCGGACTCTAAGGa                                                                                                                                                                                                                                                                                                            <  1:283824/301‑1 (MQ=255)`  
          `taaGAAACAGATGAAAGCATGGACGAAAGCTCAGGCAAAGGCTCTGGACGATTCCAGGAAGTCAATGAAAAAACTTGACGTAATCGACAAGCAATTCCAGAAGCGAATCAACGGTGAGTGGGTCTCAACGGATTTTAAGGATATGCCAGTCAACGAGAACACTGGTGAGTTCAAGAATAGCGATATGGTTAACTACGCCAATAAGAAGCGCGCTGAGATTGACAGTATGGACATTCCAGACGGTGCTAAGGATGCTATGAAGTTGAAGTACCTTCAAGCGGACTCTAAGGGCGGAGCATTc                                                                                                                                                                                                                                                                                                  <  1:324918/299‑1 (MQ=255)`  
            `aGAATCAGATGAACGCATGGACGAAAGCTCAGGCCAAGGCTCTGGACGATTCCATGAAGTCAATGAACAAACTTGACGTAATCGATAAGCAATTCCAGAAGCGAATCAACGGTGAGTGGGTCTCAACGGATTTTAAGGATATGCCAGTCAACGAGAACACTGGTGAGTTCAAGCATAGCGATATGGTTAACTACGCCAATAAGAAGCTCGCTGAGATTGACAGTATGGACATTCCAGACGGTGCCAAGGATGCTATGAAGTTGAAGTACCTTCAAGCGGACTCTAAGGGCGGAGCATTcct                                                                                                                                                                                                                                                                                                >  1:164043/1‑300 (MQ=255)`  
             `gAATCAGATGAACGCATGGACGAAAGCTCAGGCCAAGGCTCTGGACGATTCCATGAAGTCAATGAACAAACTTGACGTAATCGACAAGCAATTCCAGAAGCGAATCAACGGTGAGTGGGTCTCAACGGATTTTAAGGATATGCCAGTCAACGAGAACACTGGTGAGTTCAAGCATAGCGATATGGTTAACTACGCCAATAAGAAGCTCGCTGAGATTGACAGTATGGACATTCCAGACGGTGCCAAGGATGCTATGAAGTTGAAGTACCTTCAAGCGGACTCTAAGGACGGAGCATTCCGt                                                                                                                                                                                                                                                                                               <  1:196308/301‑1 (MQ=255)`  
               `aTCAGATGAACGCATGGACGAAAGCTCAGGCAAAGGCTCTGGACGATTCCATGAAGTCAATGAACAAACTTGACGTAATCGACAAGCAATTCCAGAAGCGAATCAACGGTGAGTGGGTCTCAACGGATTTTAAGGATATGCCAGTCAACGAGAACACTGGTGAGTTCAAGCATAGCGATATGGTTAACTACGCCAATAAGAAGCTCGCTGAGATTGACAGTATGGACATTCCAGACGGTGCCAAGGATGCTATGAAGTTGAAGTACCTTCAAGCGGACTCTAAGGGCGGAGCATTCCGTAc                                                                                                                                                                                                                                                                                             <  1:207961/301‑1 (MQ=255)`  
                 `cAGATGAACGCATGGACGAAAGCTCAGGCCAAGGCTCTGGACGATTCCATGAAGTCAATGAACAAACTTGACGTAATCGACAAGCAATTCCAGAAGCGAATCAACGTTGAGTGGGTCTCAACGGATTTTAAGGATATGCCAGTCAACGAGAACACTGGTGAGTTCAAGCATAGCGATATGGTTAACTACGCCAATAAGAAGCTCGCTGAGATTGACAGTATGGACATTCCAGACGGTGCCAAGGATGCTATGAAGTTGAAGTACCTTCAAGCGGACTCTAAGGACGGAGCATTCCGTACAg                                                                                                                                                                                                                                                                                           >  1:295346/1‑301 (MQ=255)`  
                        `aCGCATGGACGAAAGCTCAGGCAAAGGCTCTGGACGATTACATGAAGTCAATGAACAAACTTGACGTAATCGACAAGCAATTCCAGAGGCGAATCAACGGTGAGTGGGTCTCAACGGATTTTAAGGATATGCCAGTCAACGAGAACACTGGTGAGTTCAAGCATAGCGATATGGTTAACTACGCCAATAAGAAGCTCGCTGAGATTGACAGTATGGACATTCCAGACGGTGCCAAGGATGCTATGAAGTTGAAGTACCTTCAAGCGGACTCTAAGGACGGAGCATTCCGTACAGCCATCgg                                                                                                                                                                                                                                                                                    <  1:182397/301‑1 (MQ=255)`  
                             `aggACGAAAGCTCAGGCCAAGGCTCTGGACGATTCCATGAAGTCAATGAACAAACTTGACGTAATCGACAAGCAATTCCAGAAGCGAATCAACGGTGAGTGGGTCTCAACGGATTTTAAGGATATGCCAGTCAACGAGAACACTGGTGAGTTCAAGCATAGCGATATGGTTAACTACGCCAATAAGAAGCTCGCTGAGATTGACAGTATGGACATTCCAGACGGTGCCAAGGATGCTATGAAGTTGAAGTACCTTCAAGCGGACTCTAAGGGCGGAGCATTCCGTACAGCCATCGGAACCa                                                                                                                                                                                                                                                                               <  1:255751/300‑1 (MQ=255)`  
                                 `cGAAAGCTCAGGCCAAGGCTCTGGACGATTCCATGAAGTCAATGAACAAACTTGACGTAATCGACAAGCAATTCCAGAAGCGAATCAACGGTGAGTGGGTCTCAAAGGATTTTAAGGATATGCCAGTCAACGAGAACACTGGTGAGTTCAAGCATAGCGATATGGTTAACTACGCCAATAAGAAGCTCGCTGAGATTGACAGGATGGACATTCCAGACGGTGCCAAGGATGCCATGAAGTTGAAGTACCTGCAAGCGGACTCTAAGGGCGGAGCATTCCGTACAGcccctgcctcgtataa                                                                                                                                                                                                                                                                           >  1:47075/1‑287 (MQ=255)`  
                                  `aaaaGCTCAGGCCAAGGCTCTGGACGATTCCATGAAGTCAATGAACAAACTTGACGTAATCGACAAGCAATTCCAGAAGCGAATCAACGGTGAGTGGGTCTCAACGGATTTTAAGGATATGCCAGTCAACGAGAACACTGGTGAGTTCAAGCATAGCGATATGGTTAACTACGCCAATAAGAAGCTCGCTGAGATTGACAGTATGGACATTCCAGACGGTGCCAAGGATGCTATGAAGTTGAAGTACCTTCAAGCGGACTCTAAGGACGGAGCATTCCGTACAGCCATCGGAACCATGGTc                                                                                                                                                                                                                                                                          <  1:50959/300‑1 (MQ=255)`  
                                   `aaaGCTCAGGCCAAGGCTCTGGACGATTCCATGAAGTCAATGAACAAACTTGACGTAATCGACAAGCAATTCCAGAAGCGAATCAACGGTGAGTGGGTCTCAACGGATTTTAAGGATATGCCAGTCAACGAGAACACTGGTGAGTTCAAGCATAGCGATATGGTTAACTACGCCAATAAGAAGCTCGCTGAGATTGACAGTATGGACATTCCAGACGGTGCCAAGGATGCTATGAAGTTGAAGTACCTTCAAGCGGACTCTAAGGGCGGAGCATTCCGTACAGCCATCGGACCCATGGTcc                                                                                                                                                                                                                                                                         >  1:100079/1‑300 (MQ=255)`  
                                       `ataagagacaGGCTCTGGACGATTCCATGAAGTCAATGAACAAACTTGACGTAATCGACAAGCAATTCCAGAAGCGAATCAACGGTGAGTGGGTCTCAACGGATTTTAAGGATATGCCAGTCAACGAGAACACTGGTGAGTTCAAGCATAGCGATATGGTTAACTACGCCAATAAGAAGCTCGCTGAGATTGACAGTATGGACATTCCAGACGGTGCCAAGGATGCTATGAAGTTGAAGTACCTTCAAGCGGACTCTAAGGACGGAGCATTCCGTACAGCCATCGGAACCATGGTCACTGa                                                                                                                                                                                                                                                                     <  1:205257/292‑1 (MQ=255)`  
                                          `aGGCCAAGGCTCTGGACGATTCCATGAAGTCAATGAACAAACTTGACGTAATCGACAAGCAATTCCAGAAGCGAATCAACGGTGAGTGGGTCTCAACGGATTTTAAGGATATGCCAGTCAACGAGAACACTGGTGAGTTCAAGCATAGCGATATGGTTAACTACGCCAATAAGAAGCTCGCTGAGATTGACAGTATGGACATTCCAGACGGTGCCAAGGATGCTATGAAGTTGAAGTACCTTCAAGCGGACTCTAAGGGCGGAGCATTCCGTACAGCCATCGGAACCATGGTAACTGACGc                                                                                                                                                                                                                                                                  <  1:305249/301‑1 (MQ=255)`  
                                           `ggCCAAGGCTCTGGACGATTCCATGAAGTCAATGAACAAACTTGACGTAATCGACAAGCAATTCCAGAAGCGAATCAACGGTGAGTGGGTCTCAACGGATTTTAAGGATATGCCAGTCAACGAGAACACTGGTGAGTTCAAGCATAGCGATATGGTTAACTACGCCAATAAGAAGCTCGCTGAGATTGACAGTATGGACATTCCAGACGGTGCCAAGGATGCTATGAAGTTGAAGTACCTTCAAGCGGACTCTAAGGACGGAGCATGCCGTACAGCCATAGAAACCATGGTCACTGACgag                                                                                                                                                                                                                                                                 >  1:150138/1‑299 (MQ=255)`  
                                                  `gCTCTGGACGATTCCATGAAGTCAATGAACAAACTTGACGTAATCGACAAGCAATTCCAGAAGCGAATCTACGGTGAGTGGGTCTCAACGGATTTTAAGGATATGCCAGTCAAAGAGAACACTGGTGAGTTCAAGCATAGCGATATGGTTAACTACGCCAATAAGAAGCTCGCTGAGATTGACAGTATGGACATTCCAGACGGTGCCAAGGATGCTATGAAGTTGAAGTACCTTCAAGCGGACTCTAAGGACGGAGCATTCCGTACCGCCATCCGAACCATGGTCCCTGACGCTGGTCAag                                                                                                                                                                                                                                                          >  1:106470/1‑301 (MQ=255)`  
                                                  `gCTCTGGACGATTCCATGAAGTCAATGAACAAACTTGACGTAATCGACAAGCAATTCCAGAAGCGAATCAACGGTGAGTGGGTCTCAACGGATTTTAAGGATATGCCAGTCAACGAGAACACTGGTGAGTTCAAGCATAGCGATATGGTTAACTACGCCAATAAGAAGCTCGCTGAGATTGACAGTATGGACATTCCAGACGGTGCCAAGGATGCTATGAAGTTGAAGTACCTTCAAGCGGACTCTAAGGGCGGAGCATTCCGTACAGCCATCGGAACCATGGTCACTGACGCTGGTCAag                                                                                                                                                                                                                                                          >  1:264171/1‑301 (MQ=255)`  
                                                  `gCTCTGGACGATTCCATGAAGTCAATGAACAAACTTGACGTAATCGACAAGCAATTCCAGAAGCGAATCAACGGTGAGTGGGTCTCAACGGATTTTAAGGATATGCCAGTCAACGAGAACACTGGTGAGTTCAAGCATAGCGATATGGTTAACTACGCCAATAAGAAGCTCGCTGAGATTGACAGCATGGACATTCCAGACGGTGCCAAGGATGCTATGAAGTTGAAGTACCTTCAAGCGGACTCTAAGGGCGGAGCATTCCGTACAGCCATCGGAACCATGGTCACTGACGCTGGTCAag                                                                                                                                                                                                                                                          >  1:90320/1‑301 (MQ=255)`  
                                                  `gCTCTGGACGATTCCATGAAGTCAATGAACAAACTTGACGTAATCGACAAGCAATTCCAGAAGCGAATCAACGGTGAGTGGGTCTCAACGGATTTTAAGGATATGCCAGTCAACGAGAACACTGGTGAGTTCAAGCATAGCGATATGGTTAACAACGCCAATAAGAAGCTCGCTGAGATTGACAGTATGGACATTCCAGACGGTGCCAAGGATGCTATGAAGTTGAAGTACCTTCAAGCGGACTCTAAGGGCGGAGCATTCCGTACAGCCATCGGAACCATGGTCACTGACGCTGGTCAag                                                                                                                                                                                                                                                          <  1:202015/301‑1 (MQ=255)`  
                                                  `gCTCTGGACGATTCCATAAAGTCAATGAACAAACTTGACGTAATCGACAAGCAATTCCAGAAGCGAATCAACGGTGAGTGGGTCTCAACGGATTTTAAGGATATGCCAGTCAACGAGAACACTGGTGAGTTCAAGCATAGCGATATGGTTAACTACGCCAATAAGAAGCTCGCTGAGATTGACAGTATGGACATTCCAGACGGTGCCAAGGATGCTATGAAGTTGAAGTACCTTCAAGCGGACTCTAAGGGCGGAGCATTCCGTACAGCCATCGGAACCATGGTCACTGACGCTGGTCAag                                                                                                                                                                                                                                                          >  1:40413/1‑301 (MQ=255)`  
                                                    `agagacagATTCCATGAAGTCAATGAACAAACTTGACGTAATCGACAAGCAATTCCAGAAGCGAATCAACGGTGAGTGGGTCTCAACGGATTTTAAGGATATGCCAGTCAACGAGAACACTGGTGAGTTCAAGCATAGCGATATGGTTAACTACGCCAATAAGAAGCTCGCTGAGATTGACAGTATGGACATTCCAGACGGTGCCAAGGATGCTATGAAGTTGAAGTACCTTCAAGCGGACTCTAAGGGCAGAGCATTCCGTACAGCCATCGGAACCATGGTCACTGACGCTGGTCAagag                                                                                                                                                                                                                                                        <  1:130926/294‑1 (MQ=255)`  
                                                       `ggACGATTCCATGAAGTCAATGAACAAACTTGACGTAATCGACAAGCAATTCCAGAAGCGAATCAACGGTGAGTGGGTCTCAACGGATTTTAAGGATATGCCAGTCAACGAGAACACTGGTGAGTTCAAGCATAGCGATATGGTTAACTACGCCAATAAGAAGCTCGCTGAGATTGACAGTATGGACATTCCAGACGGTGCCAAGGATGCTATGAAGTTGAAGTACCTTCAAGCGGACTCTAAGGACGGAGCATTCCGTACAGCCATCGGAACCATGGTCACTGACGCTGGTCAAGAGTgg                                                                                                                                                                                                                                                     <  1:53133/301‑1 (MQ=255)`  
                                                       `ggACGATTCCATGAAGTCAATGAACAAACTTGACGTAATCGACAAGCAATTCCAGAAGAGAATCAACGGTGAGTGGGTCTCAACGGATTTTAAGGATATGCCAGTCAACGAGAACACTGGTGAGTTCAAGCATAGCGATATGGTTAACTACGCCAATAAGAAGCTCGCTGAGATTGACAGTATGGACATTCCAGACGGTGCCAAGGATGCTATGAAGTTGAAGTACCTTCAAGCGGACTCTAAGGGCGGAGCATTCCGTACAGCCATCGGAACCATGGTCACTGACGCTGGTCAagagctt                                                                                                                                                                                                                                                     >  1:155074/1‑298 (MQ=255)`  
                                                                      `gTCAATGAACAAACTTGACGTAATCGACAAGCAATTCCAGAAGCGAATCAACGGTGAGTGGGTCTCAACGGATTTTAAGGATATGCCAGTCAACGAGAACACTGGTGAGTTCAAGCATAGCGATATGGTTAACTACGCCAATAAGAAGCTCGCTGAGATTGACAGTATGGACATTCCAGACGGTGCCAAGGATGCTATGAAGTTGAAGTACCTTCAAGCGGACTCTAAGGGCGGAGCATTCCGTACAGCCATCGGAACCATGGTCACTGACGCTGGTCAAGAGCTGTCtcttatacacatc                                                                                                                                                                                                                                      >  1:125855/1‑289 (MQ=255)`  
                                                                         `aaTGAACAAACTTAACGTAATCGACAAGCAATTCCAGAAGAGAATCAACGGTGAGTGGGTCTCAACGGATTTTAAGGATATGCCAGTCAACGAGAACACTGGTGAGTTCAAGCATAGCGATATGGTTAACTACGCCAATAAGAAGCTCGCTGAGATTGACAGTATGGACATTCCAGACGGTGCCAAGGATGCTATGAAGTTGAAGTACCTTCAAGCTGACTCTAAGTGCGTAGCATTCCGTACAGCCATCGGAACCATGGTCACTGACGCTGGTCAAGAGTGGTCTGCCGCTGTGATTAAc                                                                                                                                                                                                                                   <  1:80828/301‑1 (MQ=255)`  
                                                                           `agAACAAACTTGACGTAATCGACAAGCAAGTCCAGAAGCGAAGCAACGGTGGATGGGTCTCAACGGATGTTAAGGATATGCCAGTCAACGAGAACACTGGTGAGTTCAAGCATAGCGATATGGTTAACTACGCCAATAAGAAGCTCGCTGAGATTGACAGTATTGACATTCCAGACGGTGCCAAGGATGCTATGAAGTTGAAGTACCTTCAAGCGGACTCTAAGGGCGGAGCATTCCGTACAACCATCGGAACCATGGTCACTGACGCTGGTCAAGAGTGGTCTGCCGCTGTGATTAACgg                                                                                                                                                                                                                                 <  1:64432/300‑1 (MQ=255)`  
                                                                               `cAAACTTGACGTAATCGACAAGCAATTCCAGAAGCGAATCAACGGTGAGTGGGTCTCAACGGATTTTAAGGATATGCCAGTCAACGAGAACACTGGTGAGTTCAAGCATAGCGATATGGTTAACTACGCCAATAAGAAGCTCGCTGAGATTGACAGTATGGACATTCCAGACGGTGCCAAGGATGCTATGAAGTTGAAGTACCTTCAAGCGGACTCTAAGGACGGAGCATTCCGTACAGCCATCGGAACCATGGTCACTGACGCTGGTCAAGAGTGGTCTGCCGCTGTGATTAACGGTAAg                                                                                                                                                                                                                             <  1:109684/301‑1 (MQ=255)`  
                                                                                 `aaCTTTACGTAATCGACAAGCAATTCCAGAAGCGAATCAACGGTGAGTGGGTCTCAACGGATTTTAAGGATATGCCAGTCAACGAGAACACTGGTGAGTTCAAGCATAGCGATATGGTTAACTACGCCAATAAGAAGCTCGCTGAGATTGACAGTATGGACATTCCAGACGGTGCCAAGGATGCTATGAAGTTGAAGTACCTTCAAGCGGACTCTAAGGGCGGAGCATTCCGTACAGCCATCGGAACCATGGTCACTGACGCTGGTCAAGAGTGGTCTGCCGCTGTGATTAACGGTAAGtt                                                                                                                                                                                                                           >  1:242213/1‑301 (MQ=255)`  
                                                                                              `cGACAAGCAATTCCAGAAGCGAATCAACGGTGAGTGGGTCTCAACGGATTTTAAGGATATGCCAGTCAACGAGAACACTGGTGAGTTCAAGCATAGCGATATGGTTAACTATGCCAATAAGAAGCTCGCTGAGATTGACAGTATGGACATTCCAGACGGTGCCAAGGATGCTATGAAGTTGAAGTACCTTCAAGCGGACTCTAAGGGCGGAGCATTCCGTACAGCCATCGGAACCATGGTCACTGACGCTGGTCAAGAGTGGTCTGCCGCTGTGATTAACGGTAAGTTACCAGAACGAAcc                                                                                                                                                                                                              <  1:116457/301‑1 (MQ=255)`  
                                                                                                   `aGCAATTCCAGAAGCGAATCAACGGTGAGTGGGTCTCAACGGATTTTAAGGATATGCCAGTCAACGAGAACACTGGTGAGTTCAAGCATAGCGATATGGTTAACTACGCCAATAAGAAGCTCGCTGAGATTGACAGTATGGACATTCCAGACGGTGCCAAGGATGCTATGAAGTTGAAGTACCTTCAAGCGGACTCTAAGGGCGGAGCATTCCGTACAGCCATCGGAACCATGGTCACTGACGCTGGTCAAGAGTGGTCTGCCGCTGTGATTAACGGTAAGTTACCAGAACGAAccctgtc                                                                                                                                                                                                         >  1:216685/1‑297 (MQ=255)`  
                                                                                                    `gCAATTCCAGAAGCGAATCAACGGTGAGTGGGTCTCAACGGATTTTAAGGATATGCCAGTCAACGAGAACACTGGTGAGTTCAAGCATAGCGATATGGTTAACTACGCCAATAAGAAGCTCGCTGAGATTGACAGTATGGACATTCCAGACGGTGCCAAGGATGCTATGAAGTTGAAGTACCTTCAAGCGGACTCTAAGGGCGGAGCATTCCGTACAGCCATCGGAACCATGGTCACTGACGCTGGTCAAGAGTGGTCTGCCGCTGTGATTAACGGTAAGTTAccctgtctcttatccaca                                                                                                                                                                                                        >  1:321113/1‑285 (MQ=255)`  
                                                                                                     `cAATTCCAGAAGCGAATCAACGGTGAGTGGGTCTCAACGGATTTTAAGGATATGCCAGTCAACGAGAACACTGGTGAGTTCAAGCATAGCGATATGGTTAACTACGCCAATAAGAAGCTCGCTGAGATTGACAGTATGGACATTCCAGACGGTGCCAAGGATGCTATGAAGTTGAAGTACCTTCAAGCGGACTCTAAGGACGGAGCATTCCGTACAGCCATCGGAACCATGGTCACTGACGCTGGTCAAGAGTGGTCTGCCGCTGTGATTAACggctgtctcttttacacatctccgagcc                                                                                                                                                                                                       >  1:236878/1‑275 (MQ=255)`  
                                                                                                       `aTTCCAGAAGCGAATCAACGGTGAGTGGGTCTCAACGGATTTTAAGGATATGCCAGTCAACGAGAACACTGGTGAGTTCAAGCATAGCGATATGGTTAACTACGCCAATAAGAAGCTCGCTGAGATTGACAGTATGGACATTCCAGACGGTGCCAAGGATGCTATGAAGTTGAAGTACCTTCAAGCGGACTCTAAGGGCGGAGCATTCCGTACAGCCATCGGAACCATGGTCACTGACGCTGGTCAAGAGTGGTCTGCCGCTGTGATTAACGGTAAGTTACCAGAACGAACCCCAGCGATg                                                                                                                                                                                                     >  1:267727/1‑301 (MQ=255)`  
                                                                                                         `tCCAGAAGCGAATCAACGGTGAGTGGGTCTCAACGGATTTTAAGGATATGCCAGTCAACGAGAACACTGGTGAGTTCAAGCATAGCGATATGGTTAACTACGCCAATAAGCAGCTCGCTGAGATTGACAGTATGGACATTCCAGACGGTGCCAAGGATGCTATGAAGTTGAAGTACCTTCAAGCGGACTCTAAGGACGGAGCATTCCGTACAGCCATCGGAACCATGGTCACTGACGCTGGTCAAGAGTGGTCTGCCGCTGTGATTAACGGTAAGTTACCAGAACGAACCCCAGCTATGGa                                                                                                                                                                                                   <  1:289691/301‑1 (MQ=255)`  
                                                                                                         `tCCAGAAGCGAATCAACGGTGAGTGGGTCTAAACGGATTTTAAGGATATGCCAGTAAACGAGAACACTGGGGAGTTCAAGTATAGCGATATGGTTAACTACGCCAATAAGAAGCTCGCTGAGATTGACAGGATGGACATTACAGACGGTGCCAAGGATGCTATGAAGTTGAAGTACCTTCAAGCGGACTCTAAGGACGGAGCATTCCGTACAGCCATCGGAACCATGGTCACTGACGCTGGTAAAGAGTGGTCTGCCGCTGTGATTAACGGTAAGTTACCAGAACGAACCCCAGCTATGGa                                                                                                                                                                                                   <  1:113004/301‑1 (MQ=255)`  
                                                                                                              `gacagAATCAAAGGTGAGTGGGTCTCAACGGATTTTAAGGATATGCCAGTCAACGAGAACACTGGTGAGTTCAAGTATAGCGATATGGTTAACTACGACAATAAGAAGCTCACTGAGATTGACAGTATGGACATTCCAGACGGTGCCAAGGATGCTATGAAGTTGAAGTACCTTCAAGCGGACTCTAAGGACGGAGCATTCCGTACAGCCATCGGAACCATGGGCACTGACGCTGGTCAAGAGTGGTCTGCCGCTGTGATTAACGGTAAGTTACCAGAACGAACCCCAGCTATGGATGctc                                                                                                                                                                                              <  1:332726/297‑1 (MQ=255)`  
                                                                                                              `aaGCGAATCAACGGTGAGTGGGTCTCAACGGATTTTAAGGATATGCCAGTCAACGAGAACACTGGTGAGTTCAAGCATAGCGATATGGTTAACTACGCCAATAAGAAGCTCGCTGAGATTGACAGTATGGACATTCCAGACGGTGCCAAGGATGCTATGAAGTTGAAGTACCTTCAAGCGGACTCTAAGGGCGGAGCATTCCGTACAGCCATCGGAACCATGGTCACTGACGCTGGTCAAGAGTGGTCTGCCGCTGTGATTAACGGTAAGTTACCAGAACGAACCCCAGCTATGGATGctc                                                                                                                                                                                              <  1:2107/301‑1 (MQ=255)`  
                                                                                                              `aaGCGAATCAACGGTGAGTGGGTCTCAACGGATTTTAAGGATATGCCAGTCAACGAGAACACTGGTGAGTTCAAGCATAGCGATATGGTTAACTACGCCAATAAGAAGCTCGCTGAGATTGACAGTATGGACATTCCAGACGGTGCCAAGGATGCTATGAAGTTGAAGTACCTTCAAGCGGACTCTAAGGGCGGAGCATTCCGTACAGCCATCGGAACCATGGTCACTGACGCTGGTCAAGAGTGGTCTGCCGCTGTGATTAACGGTAAGTTACCAGAACGAACCCCAGCTATGGATGctc                                                                                                                                                                                              <  1:309188/301‑1 (MQ=255)`  
                                                                                                                        `aCGGTGAGGGGGTCTCAACGGATTTGAAGGATATGCCAGTCAACGAGAACACTGGTGAGTTCAAGCATAGCGATATGGTTAACTACGCCAATAAGAAGCTCACTGAGATTGACAGTATGGACATTCCAGACGGTGCCAAGGATGCTATGAAGTTGAAGTACCTTCAAGCGGACTCTAAGGACGGAGCATTCCGTAAAGCCATCGGAACCATGGTCACTGACGCTGGTCAAGAGTGGTCTGCCGCTGTGATTAACGGTAAGTTACCAGAACGAACCCCAGCTATGGATGCTCTGCGCAGAAt                                                                                                                                                                                    <  1:167081/301‑1 (MQ=255)`  
                                                                                                                             `gAGTGGGTCTCAACGGATTTTAAGGAGATGCCAGTCAACGAGAACACTGGTGAGTTCAAGCATAGCGATATGGTTAACTACGCCAATAAGAAGCTCGCTGAGATTGACAGTATGGACATTCCAGACGGTGCCAAGGATGCTATGAAGTTGAAGTACCTTCAAGCGGACTCTAAGGGCGGAGCATTCCGTACAGCCATCGGAA‑CATGGTCACTGACGCTGGTCAAGAGTGGTCTGCCGCTGTGATTAACGGTAAGTTACCAGAACGAACCCCAGATATGGATGCCCTGCGCAGAATCCGCaa                                                                                                                                                                              >  1:67245/1‑301 (MQ=255)`  
                                                                                                                              `aGTGGGTCTCAACGGATTTTAAGGATATGCCAGTCAACGAGAACACTGGTGAGTTCAAGCATAGCGATATGGTTAACTACGCCAATAAGAAGCTCGCTGAGATTGACAGTATGGACATTCCAGACGGTGCCAAGGATGCTATGAAGTTGAAGTACCTTCAAGCGGACTCTAAGGGCGGAGCATTCCGTACAGCCATCGGAACCATGGTCACTGACGCTGGTCAAGAGTGGTCTGCCGCTGTGATTAACGGTAAGTTACCAGAACGAACCCCAGCTATGGATGCTCTGCGCAGAATCCGCaa                                                                                                                                                                              <  1:118800/301‑1 (MQ=255)`  
                                                                                                                                            `gATTTTAAGGATATGCCAGTCAACGAGAACACTGGTGAGTTCAAGCATAGCGATATGGTTAACTACGCCAATAAGAAGCTCGCTGAGATTGACAGTATGGACATTCCAGACGGTGCCAAGGATGCTATGAAGTTGAAGTACCTTCAAGCGGACTCTAAGGGCGGAGCATTCCGTACAGCCATCGGAACCATGGTCACTGACGCTGGTCAAGAGTGGTCTGCCGCTGTGATTAACGGTAAGTTACCAGAACGAACCCCAGCTATGGATGCTCTGCCCAGAATCCGCAATGCTGACCCTCAGt                                                                                                                                                                >  1:98075/1‑301 (MQ=255)`  
                                                                                                                                              `ttttAAGGATATGCCAGTCAACGAGAACACTGGTGAGTTCAAGCATATCGATATGGTTAACTACGCCAATAAGAAGCTCGCTGAGATTGACAGTATGGACATTCCAGACGGTGCCAAGGATGCTATGAAGTTGAAGTACCTTCAAGCGGACTCTAAGGACGGAGCATTCCGTACAGCCATCGGAACCATGGTCACTGACGCTGGTCAAGAGTGGTCTGCCGCTGTGATTAACGGTAAGTTACCAGAACGAACCCCAGCTATGGATGCTCTGCGCAGAATCCGCAATGCTGACCCTCAGTTg                                                                                                                                                              <  1:343896/301‑1 (MQ=255)`  
                                                                                                                                                   `ataagagacaggTCAACGAGAACACTGGTGAGTTCAAGCATAGCGATATGGTTAACTACGCCAATAAGAAGCTCGCTGAGATTGACAGTATGGACATTCCAGACGGTGCCAAGGATGCTATGAAGTTGAAGTACCTTCAAGCGGACTCTAAGGGCGGAGCATTCCATACAGCCATCGGAACCATGGTCACTGACGCTGGTCAAGAGTGGTCTGCCGCTGTGATTAACGGTAAGTTACCAGAACGAACCCCAGCTATGGATGCTCTGCGCAGAATCCGCAATGCTGACCCTCAGTTGATtgc                                                                                                                                                         <  1:330409/290‑1 (MQ=255)`  
                                                                                                                                                   `aaaagattccAGTAAACGAAAACACTGGTGAGTTCAAGTATAGCGATATGGTTAACTACGCCGATAAGAAGCTCGCTGAGATTGACAGTGTGGACATTCCAGACGGTGCCAAGGATGCTATGAAGTTGAAATACCTTCAAGCGGACTCTAAGGACGGAGCATTCCGTACAGCCATCGGAACCATGGTCACTGACGCTGGTCAAGAGTGGTCTGCCGCTGTGATTAACGGTAAGTTACCAGAACGAACCCCAGCGATGGATGCTCTGCGCAGAATCCGCAATGCTGACCCTCAGTTGATtgc                                                                                                                                                         <  1:219258/293‑1 (MQ=255)`  
                                                                                                                                                   `aGGATATGCCAGTCAACGAGAACACTGGTGAGTTCAAGCATAGCGATATGGTTAACTACGCCAATAAGAAGCTCGCTGAGATTGACAGTATGGACATTCCAGACGGTGCCAAGGATGCTATGAAGTTGAAGTACCTTCAAGCGGACTCTAAGGACGGAGCATTCCGTACAGCCATCGGAACCATGGTCACTGACGCTGGTCAAGAGTGGTCTGCCGCTGTGATTAACGGTAAGTTACCAGAACGAACCCCAGCTATGGATGCTCTGCGCAGAATCCGCAATGCTGACCCTCAGTTGAGtgc                                                                                                                                                         <  1:333876/301‑1 (MQ=255)`  
                                                                                                                                                    `ggATATGCCAGTCAACGAGAACACTGGTGAGTTCAAGCATAGCGATATGGTTAACTACGCCAATAAGAAGCTCGCTGAGATTGACAGTATGGACATTCCAGACGGTGCCAAGGATGCTATGATGTTGAAGTACCTTCAAGCGGACTCTAAGGGCGGAGCATTCCGTACAGCCATCGGAACCATGGTCACTGACGCTGGTCAAGAGTGGTCTGCCGCTGTGATTAACGGTAAGTTACCAGAACGAACCCCAGCTATGGATGCTCTGCGCAGAATCCGCAATGCTGACCCTCAGTTGATtgct                                                                                                                                                        >  1:139359/1‑301 (MQ=255)`  
                                                                                                                                                    `ggATATGCCAGTCAACGAGAACACTGGTGAGTTCAAGCATAGCGATATGGTTAACTACGCCAATAAGAAGCTCGCTGAGATTGACAGTATGGACATTCCAGACGGTGCCAAGGATGCTATGAAGTTGAAGTACCTTCAAGCGGACTCTAAGGGCGGAGCATTCCGTACAGCCATCGGAACCATGGTCACTGACGCTGGTCAAGAGTGGTCTGCCGCTGTGATTAACGGTAAGTTACCAGAACGAACCCCAGCTATGGATGCTCTGCGCAGAATCCGCAATGCTGACCCTCAGTTGATtgct                                                                                                                                                        >  1:61720/1‑301 (MQ=255)`  
                                                                                                                                                    `ggATATGCCAGTCAACGAGAACACTGGTGAGTTCAAGCATAGCGATATGGTTAACTACGCCAATAAGAAGCTCGCTGAGATTGACAGTATGGACATTCCAGACGGTGCCAAGGATGCTATGAAGTTGAAGTACCTTCAAGCGGACTCTAAGGACGGAGCATTCCGTACAGCCATCGGAACCATGGTCACTGACGCTGGTCAAGAGTGGTCTGCCGCTGTGATTAACGGTAAGTTACCAGAACGAACCCAAGCTATGGATGCTCTGCGCAGAATCCGCAATGCTGACCCCCAGTTGATtgat                                                                                                                                                        >  1:273657/1‑299 (MQ=255)`  
                                                                                                                                                            `cAGTCAACGAGAACACTGGTGAGTTCAAGCATAGCGATATGGTTAACTACGCCAATAAGAAGCTCGCTGAGATTGACAGTATGGACATTCCAGACGGTGCCAAGGATGCTATGAAGTTGAAGTACCTTCAAGCGGACTCTAAGGGCGGAGCATTCCGTACAGCCATCGGAACCATGGTCACTGACGCTGGTCAAGAGTGGTCTGCCGCTGTGATTAACGGTAAGTTACCAGAACGAACCCCAGCTATGGATGCTCTGCGCAGAATCCGCAATGCTGACCCTCAGTTGATTGCTGCGCtata                                                                                                                                                >  1:228264/1‑301 (MQ=255)`  
                                                                                                                                                             `aGTCAACGAGAACACTGGTGAGTTCAAGCATAGCGATATGGTTAACTACGCCAATAAGAAGCTCGCTGAGATTGACAGTATGGACATTCCAGACGGTGCCAAGGATGCTATGAAGTTGAAGTACCTTCAAGCGGACTCTAAGGGCGGAGCATTCCGTACAGCCATCGGGACCATGGTCACTGACGCTGGTCAAGAGTGGTCTGCCGCTGTGATTAACGGTAAGTTACCAGAACGAACCCCAGCTATGGATGCTCTGCGCAGAATCCGCAATGCTGACCCTCAGTTGATTGCTGCGCTATAc                                                                                                                                               <  1:142704/301‑1 (MQ=255)`  
                                                                                                                                                                    `gagaAAGCTGGGGAGTTCAAGCATAGCGATAGGGTGAACTAAGCCAAGAAGAAGCTCGCTGAGATTGACAGTATGGACATTCCAGACGGTGCCAAGGATGCTATGAAGTTGAAGTAACTTAAAGCGGACGCTAAGGGCGGAGCATTCCGTACAGCCATCGGAACCATGGTCACTGACGCTGGTCAAGAGTGGTCTGCCGCTGTGATTAACGGGAAGTTACCAGAACGAACCCCAGCTATGGATGCTCTGCGCAGAATCCGCAATGCTGACCCTCAGTTGATTGCTGCGCTATACCAAGAcc                                                                                                                                        <  1:85143/301‑1 (MQ=255)`  
                                                                                                                                                                          `acTGGTGAGTTCAAGCATAGCGATATGGTTAACTACGCCAATAAGAAGCTCGCTGAGATTGACAGTATGGACATTCCAGACGGTGCCAAGGATGCTATGAAGTTGAAGTACCTTCAAGCGGACTCTAAGGACGGAGCATTCCGTACAGCCATCGGAACCATGGTCACTGACGCTGGTCAAGAGTGGTCTGCCGCTGTGATTAACGGTAAGTTACCAGAACGAACCCCAGCTATGGATGCTCTGCGCAGAATCCGCAATGCTGACCCTCAGTTGATTGCTGCGCTATACCCAGACCAAGCTg                                                                                                                                  <  1:189557/301‑1 (MQ=255)`  
                                                                                                                                                                             `ggTGAGTTCAAGCATAGCGATATGGTTAACTACGCCAATAAGAAGCTCGCTGAGATTGACAGTATGGACATTCCAGACGGTGCCAAGGATGCTATGAAGTTGAAGTACCTTCAAGCGGACTCTAAGGACGGAGCATTCCGTACAGCCATCGGAACCATGGTCACTGACGCTGGTCAAGAGTGGTCTGCCGCTGTGATTAACGTTAAGTTACCAGAACGAACCCCAGCTATGGATGCTCTGCGCAGAATCCGCAATGCTGACCCTCAGTTGATTGCTGCGCTATAcgcctgtctcttataca                                                                                                                               >  1:347408/1‑285 (MQ=255)`  
                                                                                                                                                                                  `gTTCAAGCATAGCGATATGGTTAACTACGCCAATAAGAAGCGCGCTGAGATTGACAGTATGGACATTCCAGACGGTGCCAAGGATGCTATGAAGTTGAAGTACCTTCAAGCGGACTCTAAGGGCGGAGCATTCCGTACAGCCATCGGAACCATGGTCACTGACGCTGGTCAAGAGTGGTCTGCCGCTGTGATTAACGGTAAGTTACCAGAACGAACCCCAGCTATGGATGCTCTGCGCAGAATCCGCAATGCTGACCCTCAGTTGATTGCTGCGCTATACCCAGACCAAGCTGAGCTATTc                                                                                                                          <  1:43810/301‑1 (MQ=255)`  
                                                                                                                                                                                             `gCGATATGGTTAACTACGCCAATAAGAAGCTCGCTGAGATTGACAGTATGGACATTCCAGACGGTGCCAAGGATGCTATGAAGTTGAAGTACCTTCAAGCGGACTCTAAGGGCGGAGCATTCCGTACAGCCATCGGAACCATGGTCACTGACGCTGGTCAAGAGTGGTCTGCCGCTGTGATTAACGGTAAGTTACAAGAACGAACCCCCGCTATGGATGCTCTGCGCAGAATCCGCAATGCTGACCCTCAGTTGATTGCTTCGCTATACCCAGACCAAGATGGGCGATTCCTGACgtggtc                                                                                                               >  1:158282/1‑296 (MQ=255)`  
                                                                                                                                                                                              `cGATATGGTTAACTACGCCAATAAGAAGCTCGCTGAGATTGACAGTATGGACATTCCAGACGGTGCCAAGGATGCTATGAAGTTGAAGTACCTTCAAACGGACTCTAAGGACGGAGTATTCCGTACAGCCATCGGAACCATGGTCACTGACGCTGGTCAAGAGTGGTCTGCCGCTGTGATTAACGGTAAGTTACCAGAACGAACCCCAGCTATGGATGCTCTGCGCAGAATCCGCAATGCTGACCCTCAGTTGATTGCTGCGCTATACCCAGACCAAGCTGAGCTATTCCTGACGATGGAc                                                                                                              <  1:276876/301‑1 (MQ=255)`  
                                                                                                                                                                                                    `ggTTAACTACGCCAATAAGAAGCTCGCTGAGATTGACAGTATGGACATTCCAGACGGTGCCAAGGATGCTATGTAGTTGAACTACCTTCAAGCGGACTCTAAGGGCGGAGCATTCCGTACAGCCATCGGACCCATGGTCAATGACGCTGGCCAACAGTGGTCTGCCGCTGTGATTACCCCCAAGTTACCAGAACGAACCCCAGCTATGGATGCTCTGCGCAGAATCCGCAATGCTGCCCTTCAGTTGACTGCTGCGCTATCCCCGGACCAAGCCGAGCTCTTACttcagcatgtcatgatg                                                                                                        >  1:29277/1‑285 (MQ=255)`  
                                                                                                                                                                                                    `ggTTAACTACGCCAATAAGAAGCTCGCTGAGATTGACAGTATGGACATTCCAGACGGTGCCAAGGATGCTATGATGTTGAAGTACCTTCATGCGGACTCTAAGGGCGGAGCATTCCGTACAGCCATCGGAACCATGGTCACTGACGCTGGTCAAGAGTGGTCTGCCGCTGTGATTAACGGTAAGTTACCAGAACGAACCCCAGCTATGGATGCTCTGCGCAGAATCCGCAATGCTGACCCTCAGTTGATTGCTGCGCTATACCCAGACCAAGCTGAGCTATTCCTGACGATGGACatgatg                                                                                                        >  1:146717/1‑301 (MQ=255)`  
                                                                                                                                                                                                    `ggTTAACTACGCCAATAAGAAGCTCGCTGAGATTGACAGTATGGACATTCCAGACGGTGCCAAGGATGCTATGAAGTTGAAGTACCTTCAAGCGGACTCTAAGGACGGAGTATTCCGTACAGCCATCGGAACCCTGGTCACTGACGCTGGTCAAGAGTGGTCTGCCGCTGTGATTAACGGTAATTTACCAGAACGAACCCCAGCTATGGATGCCCTTCGCAGAATCCGCAATGCTTACCCTCAGTTGCTTGCTGCCCTATACCCAGACCAAGCTGAGCTATTCCTGACGTTGGACatgatg                                                                                                        >  1:231404/1‑301 (MQ=255)`  
                                                                                                                                                                                                    `ggTTAACTACGCCAATAAGAAGCTCGCTGAGATTGACAGTATGGACATTCCAGACGGTGCCAAGGATGCTATGAAGTTGAAGTACCTTCAAGCGGACTCTAAGGACGGAGCATTCCGTACAGCCATCGGAACCATGGTCACTGACGCTGGTCAAGAGTGGTCTGCCGCTGTGATTAACGGTAAGTTACCAGAACGAACCCCAGCTATGGATGCTCTGCGCAGAATCCGCAATGCTGACCCTCAGTTGATTGCTGCGCTATACCCAGACCAAGCTGAGCTATTCCTGACGATGGACatgatg                                                                                                        >  1:120949/1‑301 (MQ=255)`  
                                                                                                                                                                                                      `ttAACTACGCCAATAAGAAGCTCGCTGAGAGTGACAGTATGGACATTCCAGACGGTGCCAAGGATGCTATGAAGTTGAAGTACCGTCAAGCGGACTCTAAGGGCGGAGCATTCCGTACAGCCATCGGAACCATGGTCACTGACGCTGGTCAAGAGTGGTCTGCCGCTGTGATTAACGGTAAGTTACCAGAACGAACCCCAGCTATGGATGCTCTGCGCAGAATCCGCAATGCTGACCCTCAGTTGATTGCTGCGCTATACCCAGACCAAGCTGAGCTATTCCTGACGATGGACATGATGGa                                                                                                      <  1:152409/301‑1 (MQ=255)`  
                                                                                                                                                                                                                  `aTACGAAGCTCGCTGAGATTGACAGTATGGACATTCCAGACGGTGCCAAGGATGCTATGAAGTTGAAGTACCTTCAAGCGGACTCTAAGGACGGAGCATTCCGTACAGCCATCGGAACCATGGTCACTGACGCTGGTCAAGAGTGGTCTGCCGCTGTGATTAACGGTAAGTTACCAGAACGAACCCCAGCTATGGATGCTCTGCGCAGAATCCGCAATGCTTACCCTCAGTTGATTGCTGCGCTATACCCAGACCAAGCTGAGCTATTCCTGACGATGGACATGATGGACAAGCAGGGTAt                                                                                          >  1:161090/1‑301 (MQ=255)`  
                                                                                                                                                                                                                  `aTAAGAAGCTCGCTGAGATTGACAGTATGGACATTCCAGACGGTGCCAAGGATGCTATGAAGTTGAAGTACCTTCAAGCGGACTCTAAGGGCGGAGCATTCCGTACAGCCATCGGAACCATGGTCACTGACGCTGGTCAAGAGTGGTCTGCCGCTGTGATTAACGGTAAGTTACCAGAACGAACCCCAGCTATGGATGCTCTGCGCAGAATCCGCAATGCTGACCCTCAGTTGATTGCTGCGCTATACCCAGACCAAGCTGAGCTATTCCTGACGATGGACATGATGGACAAGCAGGGTAt                                                                                          <  1:176119/301‑1 (MQ=255)`  
                                                                                                                                                                                                                      `gtgggctcggagatgtgtataagagacagttCCAGACGGTGCCAAGGATGATATGAAGTTGAAGTACCTTCAAGCGGACTCTAAGGACGGAGAATTCCGTACAGCCATCGGAACCATGGTCACTGACGCTGGTCAAGAGTGGTCTGCCGCTGTGATTAACGGTAAGTTACCAGAACGAACCCCAGCTATGGATGCTCTGCGCAGAATCCGCAATGCTGACCCTCAGTTGATTGCTGCGCTATACCCAGACCAAGCTGAGCTATTCCTGACGATGGACATGATGGACAAGCAGGGTATTGAc                                                                                      <  1:191257/272‑1 (MQ=255)`  
                                                                                                                                                                                                                       `aggCTCGCTTGGATTGACAGTATGGACATTCCAGACTGTGCCAAGGATGCGATGAAGTTGAAGTACCTTCAAGCGGACGCTAAGGGCGGAGCATTCCGTACAGCCATCGGAACCATGGTCACTGACGCTGGTCAAGAGTGGTCTGCCGCTGTGATTAACGGTAAGTTACCAGAACGAACCCCAGCTATAGATGCTAGGCACAGAATCCGCAATGCTGACCCTCAGTTGATTGCTGCACTATACCCAGACCAAGCTGAGCTAGTCCGGACGATAGACATGATGGACAAGCAGGGTATTGAcc                                                                                     <  1:214307/299‑1 (MQ=255)`  
                                                                                                                                                                                                                       `aagCTCGCTGAGATTGACAGTATGGACATTCCAGACGGTGCCAAGGATGCTATGAAGTTGAAGTACCTTCAAGCGGACTCTAAGGGCGGAGCATTCCGTACAGCCATCGGAACCATGGTCACTGACGCTGGTCAAGAGTGGTCTGCCGCTGTGATTAACGGTAAGTTACCAGAACGAACCCCAGCTAAGGATGCTCTGCGCAGAATACGCAATGCTGACCCTCAGTTGATTGCTGCGCTATACCCAGACCAAGCTGAGCTATTCCTGACGATGGACATGATGGACAAGCAGGGTATTGAcc                                                                                     <  1:221741/301‑1 (MQ=255)`  
                                                                                                                                                                                                                       `aagCTCGCTGAGATTGACAGTATGGACATTCCAGACGGTGCCAAGGATGCTATGAAGTTGAAGTACCTTCAAGCGGACTCTAAGGACGGAGTATTCCGTACAGCCATCGGAACCATGGTCACTGACGCTGGTCAAGAGTCGTCTGCCGCTGTGATTAACGGTAAGTTACCAGAACGAACCCCAGCTATGGATGCTCTGCGCAGAATCCGCAATGCTGACCCTCAGTTGATTGCTGCGCTATACCCAGACCAAGCTGAGCTATTCCTGACGATGGACATGATGGACAAGCAGGGTATTGAcc                                                                                     <  1:253408/301‑1 (MQ=255)`  
                                                                                                                                                                                                                       `aagCTCGCTGAGATTGACAGTATGGACATTCCAGACGGTGCCAAGGATGCTATGAAGTTGAAGTACCTTCAAGCGGACTCTAAGGACGGAGCATTCCGTACAGCCATCGGAACCATGGTCACTGACGCTGGTCAAGAGTGGTCTGCCGCTGTGATTAACGGTAAGTTACCAGAACGAACCCCAGCTATGGATGCTCTGCGCAGAATCCGCAATGCTGACCCTCAGTTGATTGCTGCGCTATACCCAGACCAAGCTGAGCTATTCCTGACGATGGACATGATGGACAAGCAGGGTATTGAcc                                                                                     <  1:299116/301‑1 (MQ=255)`  
                                                                                                                                                                                                                       `aagCGCGCTGAGATTGACAGTATGGACAGTCCAGACGGTGCCAAGGATGCTATGAAGTTGAAGTACCTTCAAGCGGACTCTAAGGGCGGAGCATTCCGTACAGCCATCGGAACCATGGTCACTGACGCTGGTCAAGAGTGGTCTGCCGCTGTGATTAACGGTAAGTTACCAGAACGAACCCCAGCTATGGATGCTCTGCGCAGAATCCGCAATGCTGACCCTCAGTTGATTGCTGCGCTATACCCAGACCAAGCTGAGCTATTCCTGACGATGGACATGATGGACAAGCAGGGTATTGAcc                                                                                     <  1:10429/301‑1 (MQ=255)`  
                                                                                                                                                                                                                            `cGCTGAGATTGACAGTATGGACATTCCAGACGGTGCCAAGGATGCTATGAAGTTGAAGTACCTTCAAGCGGACTCTAAGGACGGAGCATTCCGTACAGCCATCGGAACCATGGTCACTGACGCTGGTCAAGAGTGGTCTGCCGCTGTGATTAACGGTAAGTTACCAGAACGAACCCCAGCTATGGATGCTCTGCGCAGAATCCGCAATGCTGACCCTCAGTTGATTGCTGCGCTATACCCAGACCAAGCTGAGCTATTCCTGACGATGGACATGATGGACAAGCAGGGTATTGACCCTCAg                                                                                >  1:18582/1‑301 (MQ=255)`  
                                                                                                                                                                                                                              `cTGAGATTGACAGTAGTGACATTCCATACGGTGCCAATGATGCTATGAAGTTGAAGTACCTTCAAGCGGACTCTAAGTACGGGGCATTCCGTACAGCCATCGGAACCATAGTCACTGACGCTGGTCAAGAGTGGTCTGCCGCTGTGATTAACGGTAAGTTACCAGAACGAACCCCAGCTATGGATGCTCTGCGCAGAATCCGCAATGCTGACCCTCAGTTGATTGCTGCGCTATACCCAGACCAAGCTGAGCTATTCCTGACGATGGACATGATGGACAAGCAGGGTATTGACCCTCAGGt                                                                              <  1:225073/301‑1 (MQ=255)`  
                                                                                                                                                                                                                                          `gTATGGACATTCCAGACGGTGCCAAGGATGCTATGAAGTTGAAGTACCTTCAAGCGGACTCTAAGGGCGGAGCATTCCGTACAGCCATCGGAACCATGGTCACTGACGCTGGTCAAGAGTGGTCTGCCGCTGTGATTAACGGTAAGTTACCAGAACGAACCCCAGCTATGGATGCTCTGCGCAGAATCCGCAATGCTGACCCTCAGTTGATTGCTGCGCTATACCCAGACCAAGCTGAGCTATTCCTGACGATGGACATGATGGACAAGCAGGGTATTGACCCTCAGGTTATTCTTGAttc                                                                  >  1:74114/1‑299 (MQ=255)`  
                                                                                                                                                                                                                                                   `ttCCAGACGGTGCCAAGGATGCTATGAAGTTGAAGTACCTTCAAGCGGACTCTAAGGACGGAGCATTCCGTACAGCCATCGGAACCATGGTCACTGACGCTGGTCAAGAGTGGTCTGCCGCTGTGATTAACGGTAAGTTACCAGAACGAACCCCAGCTATGGATGCTCTGCGCAGAATCCGCAATGCTGACCCTCAGTTGATTGCTGCGCTATACCCAGACCAAGCTGAGCTATTCCTGACGATGGACATGATGGACAAGCAGGGTATTGACCCTCAGGTTATTCTTGATGCCGACCgact                                                         >  1:302810/1‑301 (MQ=255)`  
                                                                                                                                                                                                                                                      `cAGACGGTGCCAAGGATGCTATGAAGTTGAAGTACCTTCAAGCGGACTCTAAGGGCGGAGCATTCCGTACAGCCATCGGAACCATGGTCACTGACGCTGGTCAAGAGTGGTCTGCCGCTGTGATTAACGGTAAGTTACCAGAACGAACCCCAGCTATGGATGCTCTGCGCAGAATCCGCAATGCTGACCCTCAGTTGATTGCTGCGTTATACCCAGACCAAGCTGAGCTATTCCTGACGATGGACATGATGGACAAGAAGGGTATTGACCCTCAGGTTATTCTTGATGCCGACCgactgac                                                      <  1:171062/301‑1 (MQ=255)`  
                                                                                                                                                                                                                                                      `cAGACGGTGCCAAGGATGCTATGAAGTTGAAGTACCTTCAAGCGGACTCTAAGGGCGGAGCATTCCGTACAGCCATCGGAACCATGGTCACTGACGCTGGTCAAGAGTGGTCTGCCGCTGTGATTAACGGTAAGTTACCAGAACGAACCCCAGCTATGGATGCTCTGCGCAGAATCCGCAATGCTGACCCTCAGTTGATTGCTGCGCTATACCCAGACCAAGCTGAGCTATTCCTGACGATGGACATGATGGACAAGCAGGGTATTGACCCTCAGGTTATTCTTGATGCCGACCgactgac                                                      <  1:271902/301‑1 (MQ=255)`  
                                                                                                                                                                                                                                                      `cAGACGGTGCCAAGGATGCTATGAAGTTGAAGTACCTTCAAGCGGACTCTAAGGGCGGAGCATTCCGTACAGCCATCGGAACCATGGTCACTGACGCTGGTCAAGAGTGGTCTGCCGCTGTGATTAACGGTAAGTTACCAGAACGAACCCCAGCTATGGATGCTCTGCGCAGAATCCGCAATGCTGACCCTCAGTTGATTGCTGCGCTATACCCAGACCAAGCTGAGCTATTCCTGACGATGGACATGATGGACAAGCAGGGTATTGACCCTCAGGTTATTCTTGATGCCGACCgactgac                                                      <  1:313508/301‑1 (MQ=255)`  
                                                                                                                                                                                                                                                            `gTGCCAAGGATGCTATGAAGTTGAAGTACCTTCAAGCGGACTCTAAGGGCGGAGCATTCCGTACAGCCATCGGAACCATGGTCACTGACGCTGGTCAAGAGTGGTCTGCCGCTGTGATTAACGGTAAGTTACCAGAACGAACCCCAGCTATGGATGCTCTGCGCAGAATCCGCAATGCTGACCCTCAGTTGATTGCTGCGCTATACCCAGACCAAGCTGAGCTATTCCTGACGATGGACATGATGGACAAGCAGGGTATTGATCCTCAGGTTATTCTTGATGCCGACCGACTGACTGTTaa                                                >  1:259932/1‑301 (MQ=255)`  
                                                                                                                                                                                                                                                            `gTGCCAAGGATGCTATGAAGTTGAAGTACCTTCAAGCGGACTCTAAGGGCGGAGCATTCCGTACAGCCATCGGAACCATGGTCACTGACGCTGGTCAAGAGTGGTCTGCCGCTGTGATTAACGGTAAGTTACCAGAACGAACCCCAGCTATGGATGCTCTGCGCAGAATCCGCAATGCTGACCCTCAGTTGATTGCTGCGCTATACCCAGACCAAGCTGAGCTATTCCTGACGATGGACATGATGGACAAGCAGGGTATTGACCCTCAGGTTATTCTTGATGCCGACCGACTGACTGTTaa                                                >  1:244652/1‑301 (MQ=255)`  
                                                                                                                                                                                                                                                            `gTGCCAAGGATGCTATGAAGTTGAAGTACCTTCAAGCGGACTCTAAGGGCGGAGCATTCCGTACAGCCATCGGAACCATGGTCACTGACGCTGGTCAAGAGTGGTCTGCCGCTGTGATTAACGGTAAGTTACCAGAACGAACCCCAGCTATGGATGCTCTGCGCAGAATCCGCAATGCTGACCCTCAGTTGATTGCTGCGCTATACCCAGACCAAGCTGAGCTATTCCTGACGATGGACATGATGGACAAGCAGGGTATTGACCCTCAGGTTATTCTTGATGCCGACCGACTAACTGTTaa                                                >  1:70663/1‑301 (MQ=255)`  
                                                                                                                                                                                                                                                             `tGCCAATGATGCTATGAAGTTGAAGTACCGTCAAGCGGACTCTAAGGACGGAGCATTCCGTACAGCCATCGGAACCATGGTCACTGACGCTGGTCAAGAGTGGTCTGCCGCTGTGATTAACGGTAAGTTACCAGAACGAACCCCAGCTATGGATGCTCTGCGCAGAATCCGCAATGCTGACCCTCAGTTGATTGCTGCGCTATACCCAGACCAAGCTGAGCTATTCCTGACGATGGACATGATGGACAAGCAGGGTATTGACCCTCAGGTTATTCTTGATGCCGACCGACTGACTGTTAAg                                               <  1:341412/301‑1 (MQ=255)`  
                                                                                                                                                                                                                                                             `gacagaGGATGCTATGAAGTTGAAGTACCTTCAAGCGGACTCTAAGGGCGGAGCATTCCGTACAGCCATCGGAACCATGGTCACTGACGCTGGTCAAGAGTGGTCTGCCGCTGTGATTAACGGTAAGTTACCAGAACGAACCCCAGCTATGGATGCTCTGCGCAGAATACGCAATGCTGACCCTCAGTTGATTGCTGCGCTATACCCAGACCAAGCTGAGCTATTCCTGACGATGGACATGATGGACAAGCAGGGTATTGACCCTCAGGTTATTCTTGATGCCGACCGACTGACTGTTAAg                                               <  1:279928/296‑1 (MQ=255)`  
                                                                                                                                                                                                                                                              `aagagacaggCTATGAAGTTGAAGTACCTTCAAGCGGACTCTAAGGGCGGAGCATTCCGTACAGCCATCGGAACCATGGTCACTGACGCTGGTCAAGAGTGGTCTGCCGCTGTGATTAACGGTAAGTTACCAGAACGAACCCCAGCTATGGATACTCTGCGCAGAATCCGCAATGCTGACCCTCAGTTGATTGCTGCGCTATACCCAGACCAAGCTGAGCTATTCCTGACGATGGACATGATGGACAAGCAGGGTATTGACCCTCAGGTTATTCTTGATGCCGACCGACTGACTGTTAAGc                                              <  1:7791/292‑1 (MQ=255)`  
                                                                                                                                                                                                                                                                `cAAGGATGCTATGAAGTTGAAGTACCTTCAAGCGGACTCTAAGGGCGGAGCATTCCGTACAGCCATCGGAACCATCGTCACTGACGCTGGTCAAGAGTGGTCTGCCGCTGTGATTAACGGTAAGTTACCAGAACGAACCCCAGCTATGGATGCTCTGCGCAGAATCCGCAATGCTGACCCTCAGTTGATTGCTGCGCTATACCCAGACCAAGCTGAGCTATTCCTGACGATGGACATGATGGACAAGCAGGGTATTGACCCTCAGGTTATTCTTGATGCCGACCGCCTGACTGTTAAGCgg                                            >  1:75681/1‑301 (MQ=255)`  
                                                                                                                                                                                                                                                                   `ggATTCTATGAAGTTGAAGTAACTTCAAGCGGACTCTAAGGGCGGAGCATTCCGTACAGCCATCGGAACCATGGTCACTGACGCTGGTCAAGAGTGGTCTGCCGCTGTGATTAACGGTAAGTTACCAGAACGAACCCCATCTATGGATGCTCTGCGCAGAATCCGCAATGCTGACCCTCAGTTGATTGCTGCGCTATACCCAGACCAATCTGAGCTATTCCTGACGATGGACATGATGGACAAGCAGGGTATTGACCCTCAGGTTATTCTTGATGCCGACCGACTGACTGTTAAGCGGTcc                                         <  1:23431/301‑1 (MQ=255)`  
                                                                                                                                                                                                                                                                     `aTGCTATGAAGTTGCAGTACCTTCAAGCGGACTCTAAGGGCGGAGCATTCCGTACAGCCATCGGAACCATGGTCACTGACGCTGGTCAAGAGTGGTCTGCCGCTGTGATTAACGGTAAGTTACCAGAACGAACCCCAGCTATGGATGCTCTGCGCAGAATCCGCAATGCTGACCCTCAGTTGATTGCTGCGCTATACCCAGACCAAGCTGAGCTATTCCTGACGATGGACATGATGGACAAGCAGGGTATTGACCCTCAGGTTATTCTTGATGCCGACCGACTGACTGTTAAGCGGTCCaa                                       <  1:239802/301‑1 (MQ=255)`  
                                                                                                                                                                                                                                                                         `aaTGAAGTTGAAGTACCTTCAAGCGTACTCTAAGGGCGGAGCATTCCGTACAGCCATCGGAACCATGGTCACTGACGCTGGTCAAGAGTGGTCTGCCGCTGTGATTAACGGTAAGTTACCAGAACGAACCCCAGCTATGGATGCTCTGCGCAGAATCCGCAATGCTGACCCTCAGTTGATTGCTGCGCTATACCCAGACCAAGCTGAGCTATTCCTGACGATGGACATGATGGACAAGCAGGGTATTGACCCTCAGGTTATTCTTGATGCCGACCGACTGACTGTTAAGCGGTCCAAagag                                   <  1:58158/300‑1 (MQ=255)`  
                                                                                                                                                                                                                                                                                 `tgaagtACCTTCAAGCGGACTCTAAGGGCGGAGCATTCCGTACAGCCATCGGAACCATGGTCACTGACGCTGGTCAAGAGTGGTCTGCCGCTGTGATTAACGGTAAGTTACCAGAACGAACCCCAGCTATGGATGCTCTGCGCAGAATCCGCAATGCTGACCCTCAGTTGATTGCTGCGCTATACCCAGACCAAGCTGAGCTATTCCTGACGATGGACATGATGGACAAGCAGGGTATTGACCCTCAGGTTATTCTTGATGCCGACCGACTGACTGTTAAGCGGTCCAAAGAGCAACGCtt                           <  1:133861/301‑1 (MQ=255)`  
                                                                                                                                                                                                                                                                                      `ggtattagagacagctctAAGGGCGGAGCATTCCGTACAGCCATCGGAACCATGGTCACTGACGCTGGTCAAGAGTGGTCTGCCGCTGTGATTAACGGTAAGTTACCAGAACGAACCCCAGCTATTGATGCTCTGCGCAGAATCCGCAATGCTGACCCTCAGTTGATTGCTGCGCTATACCCAGACCAAGCTGAGCTATTCCTGACGATGGACATGATGGACAAGCAGGGTATTGACCCTCAGGTTATTCTTGATGCCGACCGACTGACTGTTAAGCGGTCCAAAGAGCAACGCTTTGAgg                      <  1:93955/287‑1 (MQ=255)`  
                                                                                                                                                                                                                                                                                        `ccTTCAAGCGGACTCTAAGGGCGGAGCATTCCGTACAGCCATCGGAACCATGGTCACTGACGCTGGTCAAGAGTGGTCTGCCGCTGTGATTAACGGTAAGTTACCAGAACGAACCCCAGCTATGGATGCTCTGCGCAGAATCCGCAATGCTGACCCTCAGTTGATTGCTGCGCTATACCCAGACCAAGCTGAGCTATTCCTGACGATGGACATGATGGACAAGCAGGGTATTGACCCTCAGGTTATTCTTGATGCCGACCGACTGACTGTTAAGCGGTCCAAAGAGCAACGCTTTGAGgat                    >  1:262050/1‑301 (MQ=255)`  
                                                                                                                                                                                                                                                                                        `ccTTCAAGCGGACTCTAAGGGCGGAGCATTCCGTACAGCCATCGGAACCATGGTCACTGACGCTGGTCAAGAGTGGTCTGCCGCTGTGATTAACGGTAAGTTACCAGAACGAACCCCAGCTATGGATGCTCTGCGCAGAATCCGCAATGCTGACCCTCAGTTGATTGCTGCGCTATACCCAGACCAAGCTGAGCTATTCCTGACGATGGACATGATGGACAAGCAGGGTATTGACCCTCAGGTTATTCTTGATGCCGACCGACTGACTGTTAAGCGGTCCAAAGAGCAACGCTTTGAGgat                    <  1:37372/301‑1 (MQ=255)`  
                                                                                                                                                                                                                                                                                             `aaGCGGTCTCTAAGGACGGAGCATTCCGTACAGCCATCGGAACCATGGTCACTGACGCTGGTCAAGAGTGGTGTGACGCTGGGATTAACGGTAAGTTACCAGAACGAACCCCAGCTATGGATGCTCTGCGCAGAATCCGCAAGGCTGACCCTCAGTTGATTGCTGCGCTATACCCAGACCAAGCTGAGCTATTCCTGACGATAGACATGATGGACAAGCAGGGTATTGACCCTCAGGTTATTCTTGATGCCGACCGACTGACTGTTAAGCGGTCCGAAGAGCAACGCTTTGAGGATGATaa               <  1:250651/301‑1 (MQ=255)`  
                                                                                                                                                                                                                                                                                                  `gACTCTAAGGGCGGAGCATTCCGTACAGCCATCGGAACCATGGTCACTGACGCTGGTCAAGAGTGGTCTGCCGCTGTGATTAACGGTAAGTTACCAGAACGAACCCCAGCTATGGATGCTCTGCGCAGAATCCGCAATGCTTACCCTCAGTTGATTGCTGCGCTATACCCAGACCAAGCTGAGCTATTCCTGACGATGGACATGATGGCCAAGCAGGGTATTGACCCTCAGGTTATTCTTGATGCCGACCGACTGACTGTTAAGCGGTCCAAAGAGCAACGCTTTGAGGTTGAAAAAGCAt          >  1:179376/1‑301 (MQ=255)`  
                                                                                                                                                                                                                                                                                                  `gACTCTAAGGGCGGAGCATTCCGTACAGCCATCGGAACCATGGTCACTGACGCTGGTCAAGAGTGGTCTGCCGCTGTGATTAACGGTAAGTTACCAGAACGAACCCCAGCTATGGATGCTCTGCGCAGAATCCGCAATGCTGACCCTCAGTTGATTGCTGCGCTATACCCAGACCAAGCTGAGCTATTCCTGACGATGGACATGATGGACAAGCAGGGTATTGACCCTCAGGTTATTCTTGATGCCGACCGACTGACTGTTAAGCGGTCCAAAGAGCAACGCTTTGAGGATGATAAAGCAt          >  1:287020/1‑301 (MQ=255)`  
                                                                                                                                                                                                                                                                                                   `aCTCTAAGGGCGGAGCAGTCCGTACAGCCATCGGAACCATGGTCACTGACGCTGGTCAAGAGTGGTCTGCCGCTGTGATTAACGGGAAGTTACCAGAACGAACCCCAGCTATGGATGCTCTGCGCAGAATCCGCAATGCTGACCCTCATTTGATTGCTGCGCTATACCCAGACCAAGCTGAGCTATTCCTGACGATGGACATGATGGACAAGCAGGGTATTGACCCTCAGGTTATTCTTGATGCCGACCGACTGACTGTTAAGCGGTCCAAAGAGCAACGCTTTGAGGATGATAAAGCAtt         <  1:336352/301‑1 (MQ=255)`  
                                                                                                                                                                                                                                                                                                    `ctctAAGGACGGCGCCTTCCGTACAGCCATCGGAACCATGGTCACTGACGCTGGTCAAGAGTGGTCTGCCGCTGTGATTAACGGTAAGTTACCCAAACGAACCCCAGCTATTGATGCTCTGCGCAGAATCCGCAATGCTTACCCTCAGTTGATTGCTGCGCTATACCCAGACCAAGCTGAGCTATTCCTGACGATGGACATGATGGACAAGCAGGGTATTTACCCTCAGGTTATTCTTGATGCCGACCGACTGACTGTTAAGCGGTCCAAAGAGAAACGCATTGAGGATGATAAAGCATTc        >  1:56028/1‑301 (MQ=255)`  
                                                                                                                                                                                                                                                                                                     `tctAAGGGCGGAGCATTCCGTACAGCCATCGGAACCATGGTCACTGACGCTGGTCAAGAGTGGTCTGCCGCTGTGATTAACGGTAAGTTACCAGAACGAACCCCAGCTATGGATGCTCTGCGCAGAATCCGCAATGCTGACCCTCAGTTGATTGCTGCGCTATACCCAGACCAAGCTGAGCTATTCCTGACGATGGACATGATGGACAAGCAGGGTATTGACCCTCAGGTTATTCTTGATGCCGACCGACTGACTGTTAAGCGGTCCAAAGAGCAACGCTTTGAGGATGATAAAGCATTCg       <  1:327356/301‑1 (MQ=255)`  
                                                                                                                                                                                                                                                                                                       `tAAGGGCGGAGCATTCCGTACAGCCATCGGAACCATGGTCACTGACGCTGGTCAAGAGTGGTCTGCCGCTGTGATTAACGGTAAGTTACCAGAACGAACCCCAGCTATGGATGCTCTGCGCAGAATCCGCAATGCTGACCCGCAGTTGATTGCTGCGCTATACCCAGACCAAGCTGAGCTATTCCTGACGATGGACATGATGGACAAGCAGGGTATTGACCCTCAGGTTATTCTTGATGCCGACCGACTGACTGTTAAGCGGTCCAAAGAGCAACGCTTTGAGGATGATAAAGCATTCGAg     <  1:137372/301‑1 (MQ=255)`  
                                                                                                                                                                                                                                                                                                         `aGGACGGAGCATTCCGTACAGCCATCGGAACCATGGTCACTGACGCTGGTCAAGAGTGGTCTGCCGCTGTGATTAACGGTAAGTTACAAGAACGAACCCCAGCTATGGATGCTCTGCGCAGAATCCGCAATGCTGACACTCAGTTGATTGCTGCGCTATACCCAGACCAAGCTGAGCTATTCCTGACGATGGACATGATGGAAAAGCAGGGTACTGACCCTCAGGTTATTCTTGATGCCGACCGACTGACTGTTAAGCGGTCCAAAGAGCAACGCTTTGAGGATGATAAAGCATTCGAGTc   <  1:316360/301‑1 (MQ=255)`  
                                                                                                                                                                                                                                                                                                          `ggACGGAGCATTCCGTACAGCCATCGGAACCATGGTCACTGACGCTGGTCAAGAGTGGTCTGCCGCTGTGATTAACGGTAAGTTACCAGAACGAACCCCAGCTATGGATGCTCTGCGCAGAATCCGCAATGCTGACCCTCAGTTGATTGTTGCGCTATACCCAGACCAAGCTGAGCTATTCCTGACGATGGACATGATGGAAAAGCAGGGTATTGACCCCCAGGTTATTCTTGATGCCGACCGACTGACTGGTAAGCGGTCCAAAGAGCAACGCCTTGAGGATGATAAAGAATTCGAGTCt  >  1:123919/1‑301 (MQ=255)`  

                                                                                                                                                                                                                                                                                                            `|`                                                                                                                                                                                                                                                                                                            
`AGGAACAAGTTCAGAATCAGATGAACGCATGGACGAAAGCTCAGGCCAAGGCTCTGGACGATTCCATGAAGTCAATGAACAAACTTGACGTAATCGACAAGCAATTCCAGAAGCGAATCAACGGTGAGTGGGTCTCAACGGATTTTAAGGATATGCCAGTCAACGAGAACACTGGTGAGTTCAAGCATAGCGATATGGTTAACTACGCCAATAAGAAGCTCGCTGAGATTGACAGTATGGACATTCCAGACGGTGCCAAGGATGCTATGAAGTTGAAGTACCTTCAAGCGGACTCTAAGGACGGAGCATTCCGTACAGCCATCGGAACCATGGTCACTGACGCTGGTCAAGAGTGGTCTGCCGCTGTGATTAACGGTAAGTTACCAGAACGAACCCCAGCTATGGATGCTCTGCGCAGAATCCGCAATGCTGACCCTCAGTTGATTGCTGCGCTATACCCAGACCAAGCTGAGCTATTCCTGACGATGGACATGATGGACAAGCAGGGTATTGACCCTCAGGTTATTCTTGATGCCGACCGACTGACTGTTAAGCGGTCCAAAGAGCAACGCTTTGAGGATGATAAAGCATTCGAGTCT  >  NC_001604.1/29391‑29989`

  

Alignment Legend

Aligned base mismatch/match (shaded by quality score): `ATCG/ATCG` `< 3 ≤` `ATCG/ATCG` `< 11 ≤` `ATCG/ATCG` `< 24 ≤` `ATCG/ATCG` `< 35 ≤` `ATCG/ATCG` `< 38 ≤` `ATCG/ATCG`

Unaligned base: `atcg`    Masked matching base: `atcg`    Alignment gap: `‑`    Deleted base: `‑`
